# The prognostic value and immune correlation of IL18 expression and promoter methylation in renal cell carcinoma

**DOI:** 10.1186/s13148-023-01426-8

**Published:** 2023-01-28

**Authors:** Xiaonan Wang, Wancui Zhu, Qian Long, Enni Chen, Haohui Sun, Xiaodi Li, Hailin Xu, Weizhao Li, Pei Dong, Liru He, Miao Chen, Wuguo Deng

**Affiliations:** 1 Sun Yat-Sen University Cancer Center, State Key Laboratory of Oncology in South China, Collaborative Innovation Center of Cancer Medicine, Guangzhou, China; 2 Zhongshan School of Medicine, Sun Yat-Sen University, Guangzhou, China; 3 Department of General Surgery, The Second Xiangya Hospital, Central South University, Changsha, China; 4 The First Affiliated Hospital, Sun Yat-Sen University, Guangzhou, China; 5 The Seventh Affiliated Hospital, Sun Yat-Sen University, Shenzhen, China

**Keywords:** Renal cell carcinoma (RCC), IL18, DNA methylation, Epigenetic biomarker, Tumor immune cell infiltration

## Abstract

**Background:**

Renal cell carcinoma (RCC) is not sensitive to immunotherapy and has poor prognosis. DNA methylation regulates gene expression, and its abnormal changes are related to many human diseases. Recently, DNA methylation has been found to participate in immune infiltration in various cancers. However, its pattern in RCC remains poorly understood.

**Results:**

We found that IL18 was significantly over-expressed in RCC tumor tissues compared to normal adjacent tissues The IL18 promoter region was hypomethylated, which was strongly correlated with elevated IL18 mRNA expression, and predicted advanced clinicopathological characteristics and shorter overall survival. Furthermore, we found that IL18 promoter methylation was significantly related to the down-regulation of immune checkpoint molecules and increase of CD8 + T cell infiltration in RCC tumor tissues.

**Conclusions:**

We have identified the important role of IL18 promoter methylation and expression, which are associated with clinicopathological characteristics, overall survival, immune cell infiltration and expression of immune checkpoint molecules in RCC. We present the rationale for IL18 promoter methylation as a molecular biomarker for predicting the response of RCC to immune checkpoint inhibitors.

**Supplementary Information:**

The online version contains supplementary material available at 10.1186/s13148-023-01426-8.

## Background

IL18 was early found as an endotoxin-induced serum factor [1]. It is one of the members of the IL1 family, which is produced from a 24Kda precursor and then cleaved into its mature form by caspase-1 [2]. Most normal cells in human, especially macrophages and dendritic cells, can produce IL18. Moreover, IL18 can induce NK cells to produce INFγ [3, 4]. Recently more and more studies have focused on the effects of IL18 on cancer. Interestingly, depending on cellular context, IL18 can be an anti-tumoral factor or an oncogenic factor. On the other hand, studies have shown that after normal intestinal epithelial cells become cancerous, they no longer produce IL18, resulting in down-regulation of IFN levels, which in turn allows cancer cells to escape from immune surveillance [5].

On the other hand, studies have shown that IL18 can increase the toxicity of NK cells by up-regulating the expression of Fas ligands on T cells [6]. In addition, IL18 can stimulate systemic inflammation and local inflammation by inducing the production of TNFα, COX2, GM-CSF, etc. [7, 8]. IL-18 can also promote liver metastasis of melanoma cells by regulating the expression of vascular cell adhesion molecule-1 and the adhesion of melanoma cells [9]. Studies have shown that IL18 is highly expressed in the serum of gastric cancer patients, and endogenous IL18 can promote the metastasis of gastric cancer by enhancing the expression of CD44 and VGEF and inhibiting the expression of CD70 [10]. A large number of studies have shown that IL18 has obvious anti-tumor effects in mouse tumor models and clinical experiments [11–13].

Renal cell carcinoma (RCC) is the most common renal malignancy, and its incidence is significantly higher in men than in women [14, 15]. The incidence of RCC accounts for 3% of all cancers, especially in Western countries [14, 15]. According to histological classification, RCC can be categorized into three main types: clear cell renal cell carcinoma (ccRCC, 70–80%), papillary renal cell carcinoma (pRCC), and chromophobe renal cell carcinoma (4–5%). There are differences in tumor stage, grade and cancer-specific survival (CSS) among RCC subtypes, which have prognostic implications [14, 15]. Immunotherapy has made great progress in recent years, and studies have shown that renal cell carcinoma is highly immunogenic and sensitive to immunotherapy [16–18]. However, there are still some RCC patients who already have metastasis when initially diagnosed, and the effect of immunotherapy is limited in them [19], the survival rate of such patients is lower compared to others. As the overall prognosis is poor in RCC patients, it is particularly important to develop new strategies to improve the efficacy of immunotherapy in RCC.

Accumulating evidence has shown that abnormal changes in DNA methylation are related to many human diseases [20]. DNA methylation is involved in the regulation of gene expression and widely exists in immune cells [21]. It has been proved that abnormal intracellular methylation levels may lead to cancerous changes in cells [22]. Methylation of certain promoter sites may be reliable biomarkers for predicting immune checkpoint inhibitor response [23]. Studies have shown that promoter methylation of the inflammatory factor NLRP3 is associated with favorable prognosis and decreased immune infiltration in RCC [24].

So far, the researches on the role of IL18 in tumors have mainly focused on the promotion or inhibition of tumor development or immune infiltration mediated by IL18 through regulating related signaling pathways. However, it remains unknown the effect of IL18 promoter methylation on the occurrence, development and immune infiltration of tumors, especially renal cell carcinoma. The purpose of this study was to explore the status of IL18 promoter methylation in RCC and its effect on the occurrence, development and immune infiltration of RCC.

## Results

### IL18 was highly expressed and associated with poor prognosis in RCC patients

**Fig. 1:**
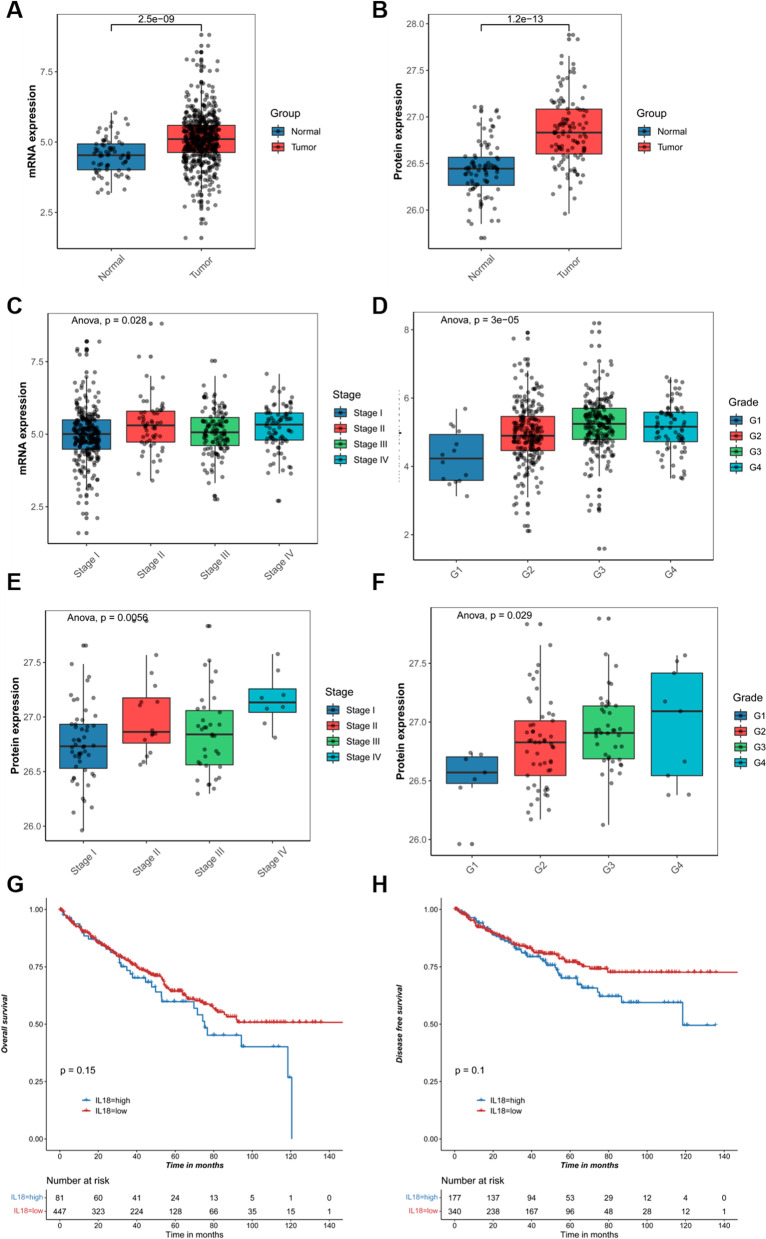
IL18 is highly expressed and associated with poor prognosis in RCC patients. **A**. Relative mRNA expression of IL18 between tumor and adjacent normal tissues using the data from TCGA cohort; **B** Relative expression of IL18 between tumor and adjacent normal tissues using the data from CPTAC cohort; **C**–**F** The expression of IL18 expression in different stages and pathological grades of patients from TCGA cohort; **G**, **H** Kaplan–Meier survival analysis of IL18 expression for overall survival and disease-free survival of patients from TCGA RCC cohort, respectively

IL18 is an anti-tumoral factor or an oncogenic factor in different type of cancers. To investigate the role of IL18 in RCC, we firstly analyzed the expression of IL18 in tumor tissues and paired normal adjacent tissues. We found IL18 was highly expressed in RCC tumor tissues compared to normal tissues (Fig. 1A, B). Furthermore, we also found that the expression of IL18 was differentially expressed in renal cells with different pathological grades and GRADE grades, and it was positively correlated with the grade (Fig. 1C–F). By analyzing the prognostic information of RCC patients in the TCGA database, we found that the higher expression of IL18 was associated with the shorter overall survival and disease-free survival of patients (Fig. 1G, H). These results demonstrated that IL18 was highly expressed and associated with poor prognosis in RCC patients.

### IL18 promoter was abnormally methylated in RCC

**Fig. 2:**
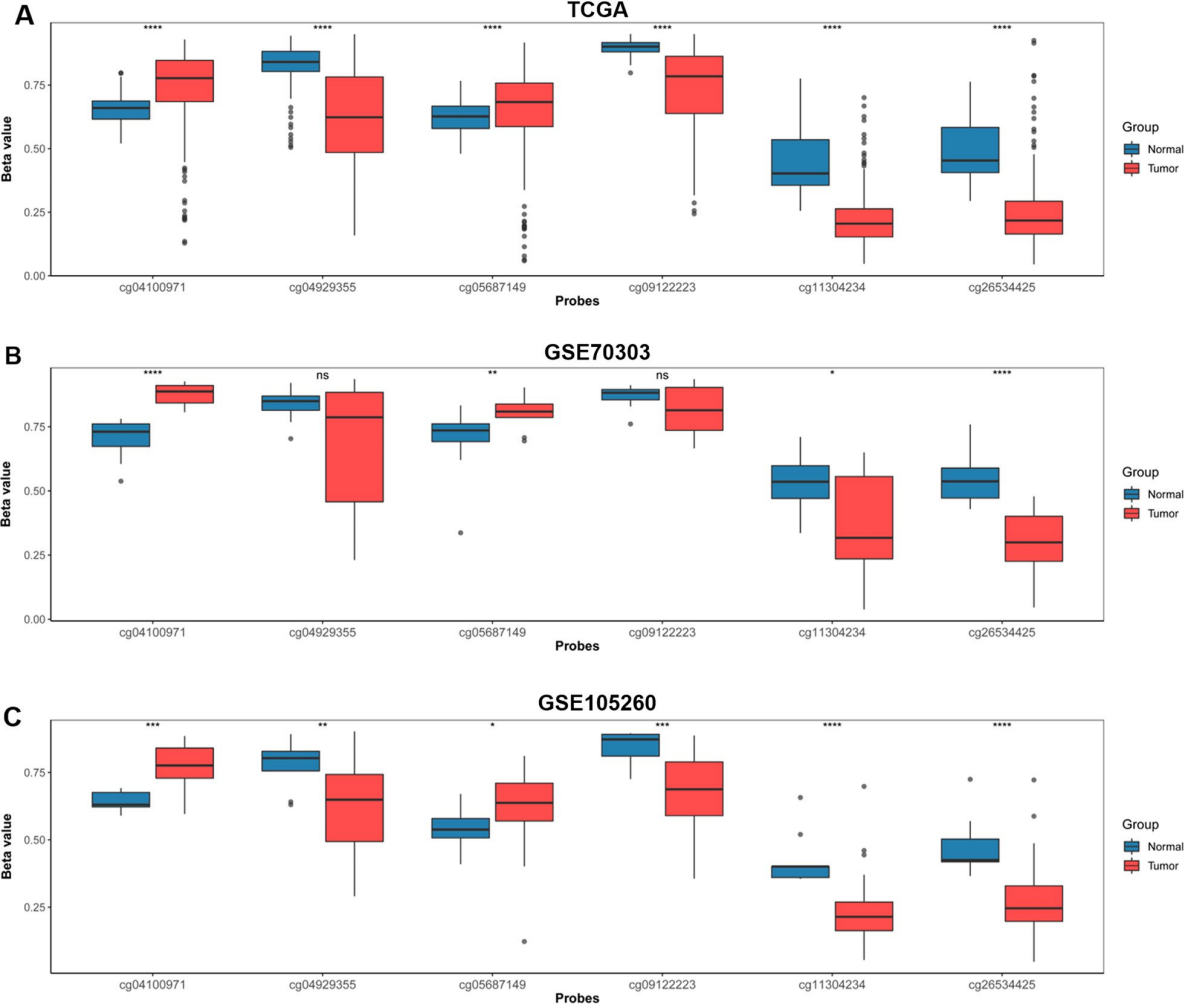
IL18 promoter was abnormally methylated in tumor versus normal adjacent tissues in RCC. **A**–**C** Relative methylation of the six differentially methylated CpG sites (cg04100971, cg04929355, cg05687149, cg09122223, cg11304234 and cg26534425) located in IL18 promoter between tumor and adjacent normal tissues of TCGA cohort, GSE70303 and GSE105260, respectively

**Fig. 3:**
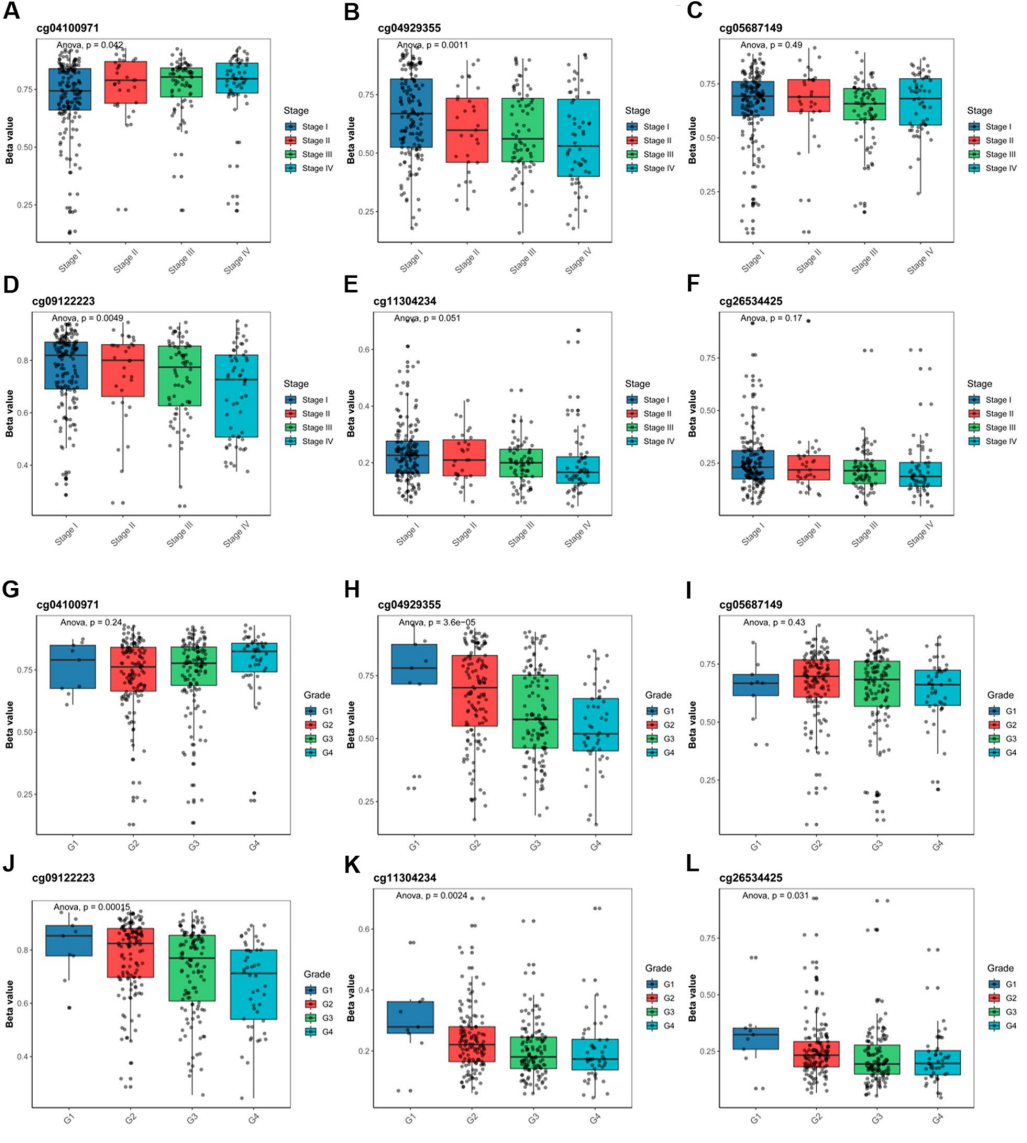
Promoter methylation was associated with aggressive clinical phenotypes in RCC. **A**–**L** Relative expression of methylation of cg04100971, cg04929355, cg05687149, cg09122223, cg11304234 and cg26534425 in different stages and pathological grade of patients from TCGA cohort

More and more researches have suggested that DNA promoter methylation plays an important role in prognosis and grading of tumors [24]. Therefore we further explored the role of IL18 promoter methylation in RCC. First of all, we used the relevant website (http://www.bioinfo-zs.com/smartapp/#) to explore the methylation condition of the IL18, and further examined the six main methylation sites: cg04100971, cg04929355, cg05687149, cg09122223, cg11304234 and cg26534425 (Additional file 1: Fig. S1). To validate IL18 promoter methylation in tumor tissues, we analyzed these 6 methylation sites in IL18 promoter in TCGA database and anther two GEO datasets. The sites of cg04100971 and cg05687149 were significantly hypermethylated in tumor compared to normal tissue. (Fig. 2A–C). We also found that the other four methylation sites were hypomethylated in tumor tissue versus normal tissue in all three or some of the datasets (Fig. 2A–C). We further explored the correlation between the methylation of IL18 promoter and the clinical characteristics of renal cancer patients, we analyzed IL18 and the six methylation sites on IL18 promoter (cg04100971, cg04929355, cg05687149, cg09122223, cg11304234 and cg26534425) with the clinicopathological data of renal cancer patients by using the TCGA data. We found that the promoter methylation level of IL18 was different in different pathological grades and stages of RCC patients. The higher the grade was, the higher the methylation levels of cg04100971 and cg05687149 were, but the lower the methylation level of the cg04929355, cg09122223, cg11304234 and cg26534425 were (Fig. 3A–K). The promoter methylation level of IL18 was also related to the gender and ages (Additional file 2: Table S7), but not associated with smoking (Additional file 2: Table S8). These results indicated that IL18 promoter was abnormally methylated in RCC and differences in methylation levels were also related to the grading of RCC.

### IL18 promoter methylation correlated with IL18 expression in RCC

**Fig. 4:**
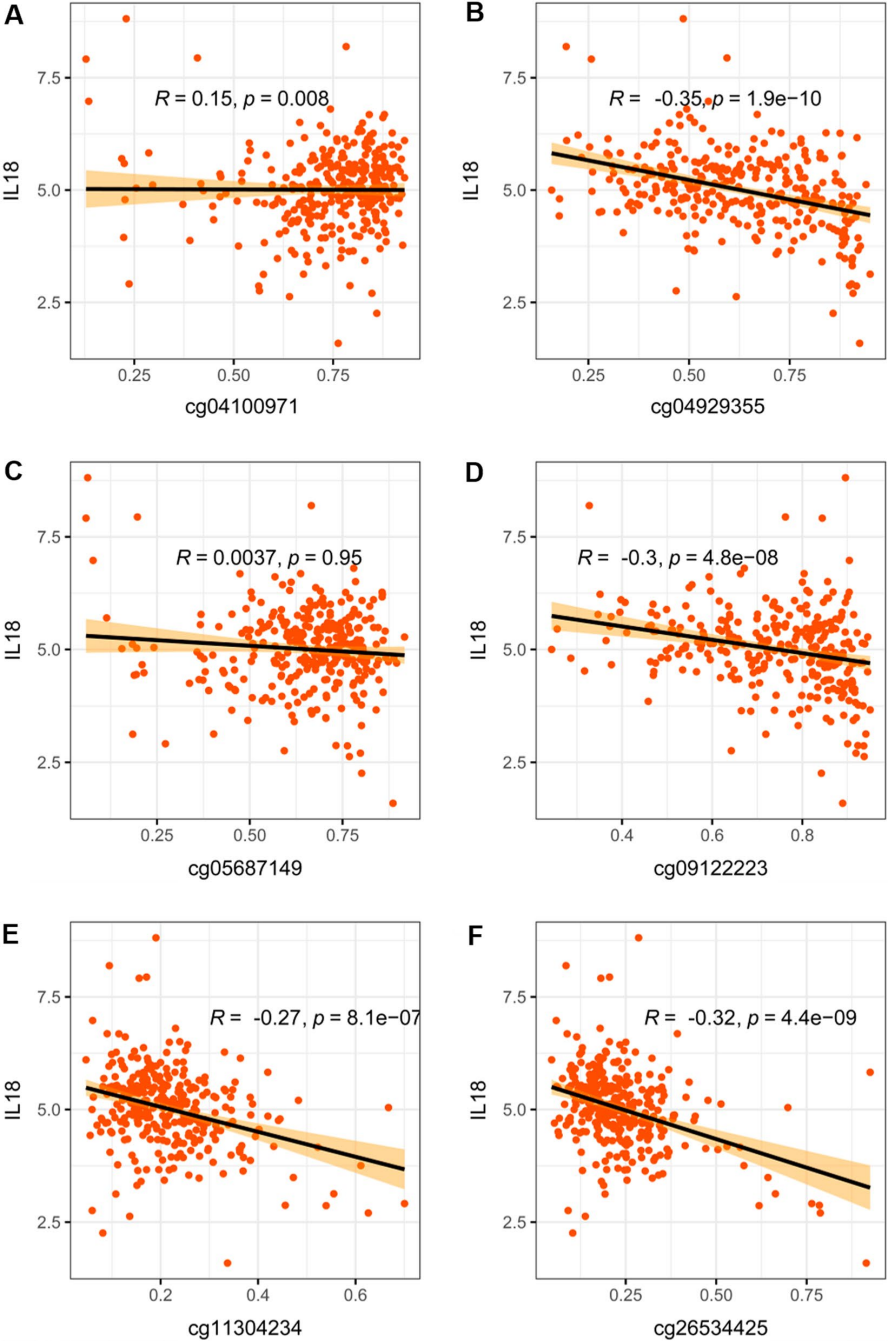
The correlation of IL18 promoter methylation with IL18 in RCC. **A**–**F** The correlation of cg04100971, cg04929355, cg05687149, cg09122223, cg11304234 and cg26534425 methylation with IL18 expression in TCGA tumor tissues

We further explored whether the expression of IL18 is related to the methylation level of IL18 promoter. To this end, we examined the IL18 expression and promoter methylation level in the TCGA database, and found that in addition to the cg05687149 site, the other five methylation sites were also related to the expression of IL18 (Fig. 4A–F). To further explore the role of IL18 promoter methylation in its expression, we used 5-Azacytidine experiments, a cytidine nucleoside analog that specifically inhibits DNA methylation, to inhibit DNA methylation in ACHN and 786-O cells. We found that IL18 expression was significantly increased after treatment with 5-Azacytidine for 72 h (Additional file 1: Fig. S2). These mean that abnormal methylation of IL18 promoter may cause the abnormal high expression of IL18 in RCC.

### The promoter methylation level of IL18 predicted prognosis in RCC

**Fig. 5:**
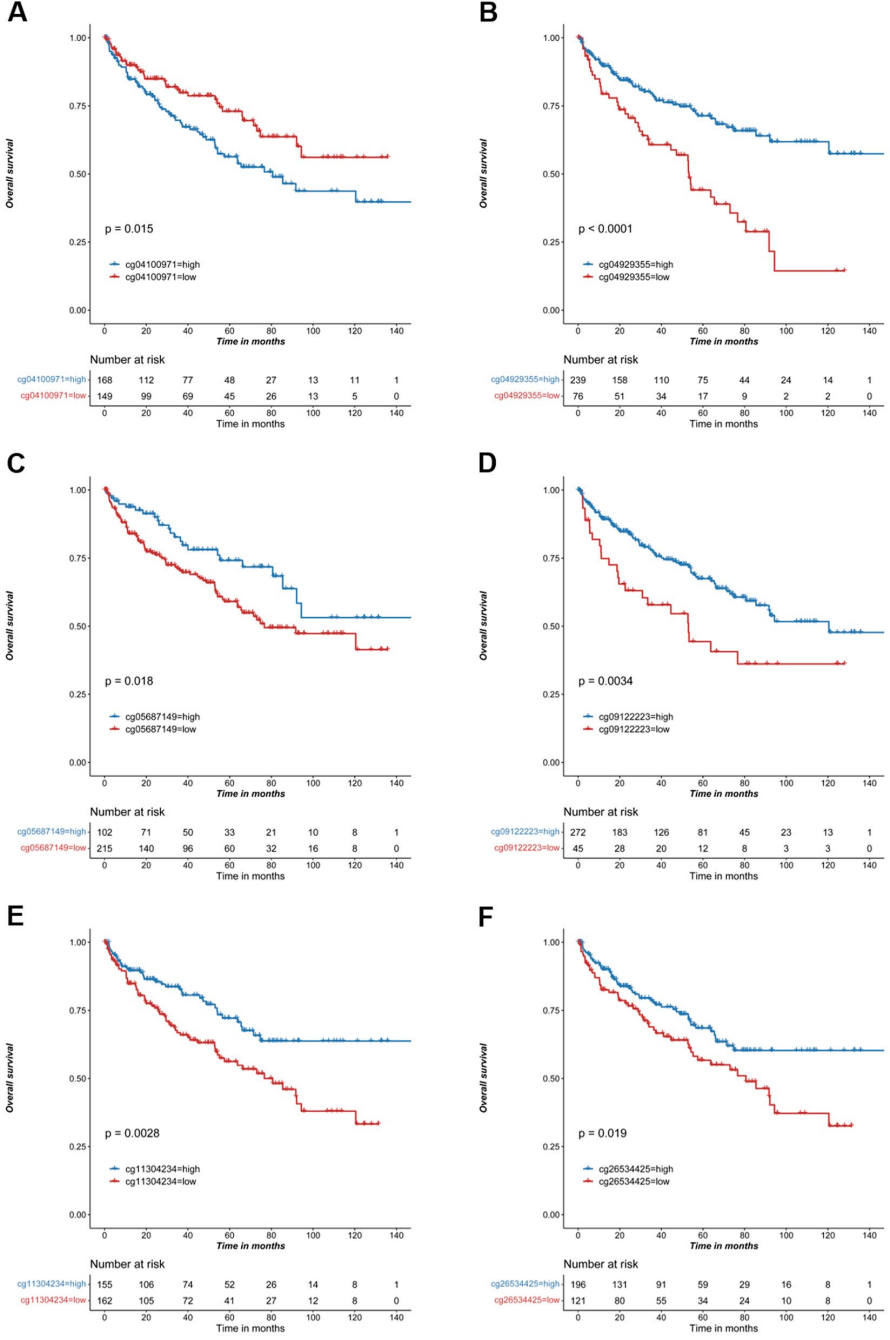
IL18 promoter methylation predicted overall survival in RCC. **A**–**F**. Kaplan–Meier survival analysis of J cg04100971, cg04929355, cg05687149, cg09122223, cg11304234 and cg26534425 for overall survival of patients from TCGA RCC cohort, respectively

**Fig. 6:**
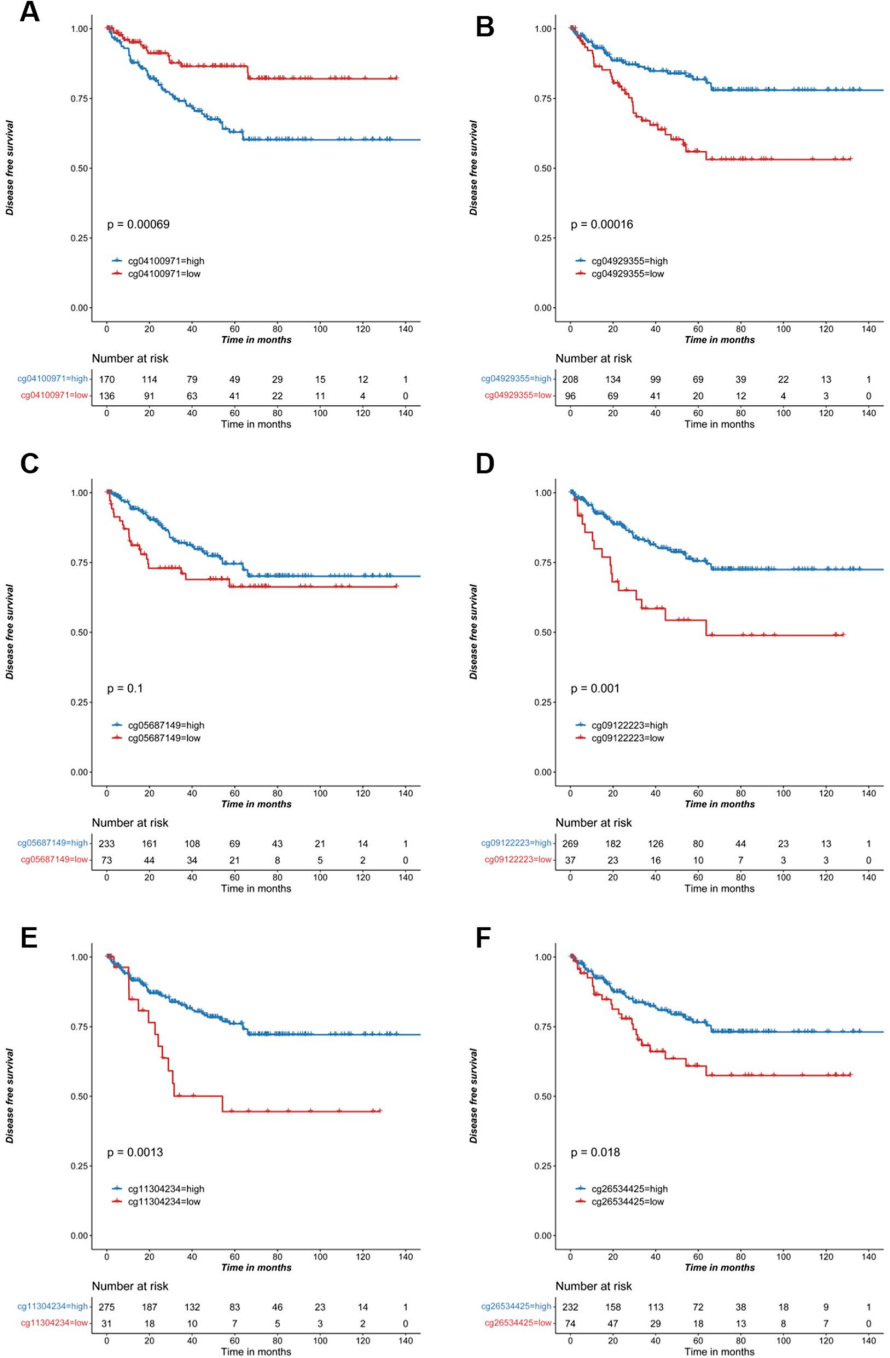
IL18 promoter methylation predicted disease-free survival in RCC. **A**–**F** Kaplan–Meier survival analysis of cg04100971, cg04929355, cg05687149, cg09122223, cg11304234 and cg26534425 for disease-free survival of patients from TCGA RCC cohort, respectively

To explore the prognostic value of IL18 promoter methylation in RCC, we first analyzed the association of these 6 methylation sites with the overall survival in RCC patients. We found that the hypermethylated site cg04100971 was associated with a poor overall survival (Fig. 5A) and disease-free survival (Fig. 6A), while another hypermethylated site cg05687149 predicted favorable overall survival (Fig. 5C). Oddly, the methylation of cg05687149 was not related to IL18 expression (Fig. 4C). We also found that the other four hypomethylated sites were associated with favorable overall (Fig. 5B, D–F) and disease-free survival (Fig. 6B, D–F). These results showed that the promoter methylation level of IL18 may be a potential biomarker in predicting prognosis in RCC.

### IL18 expression and promoter methylation were correlated with immune cell infiltration in RCC

**Fig. 7:**
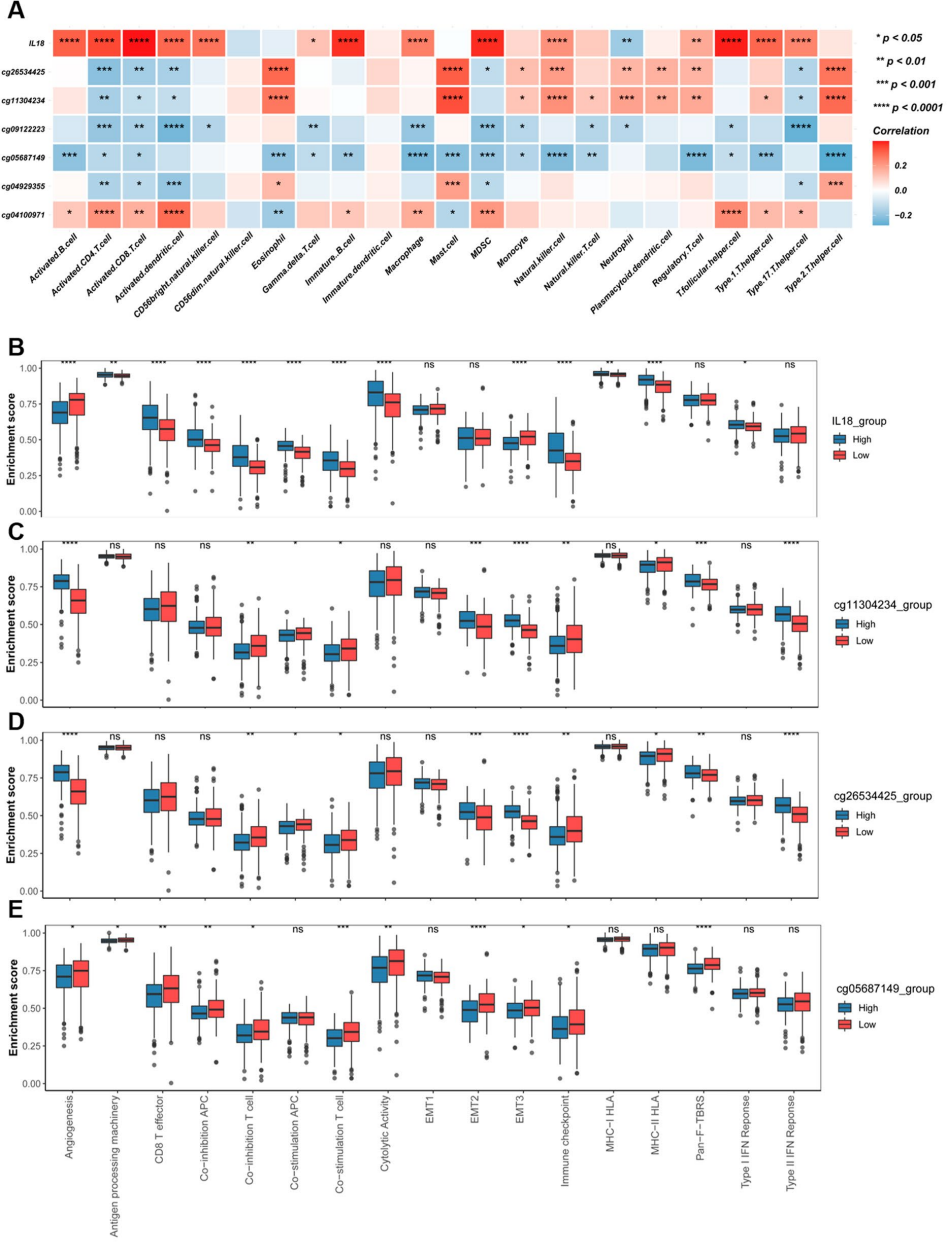

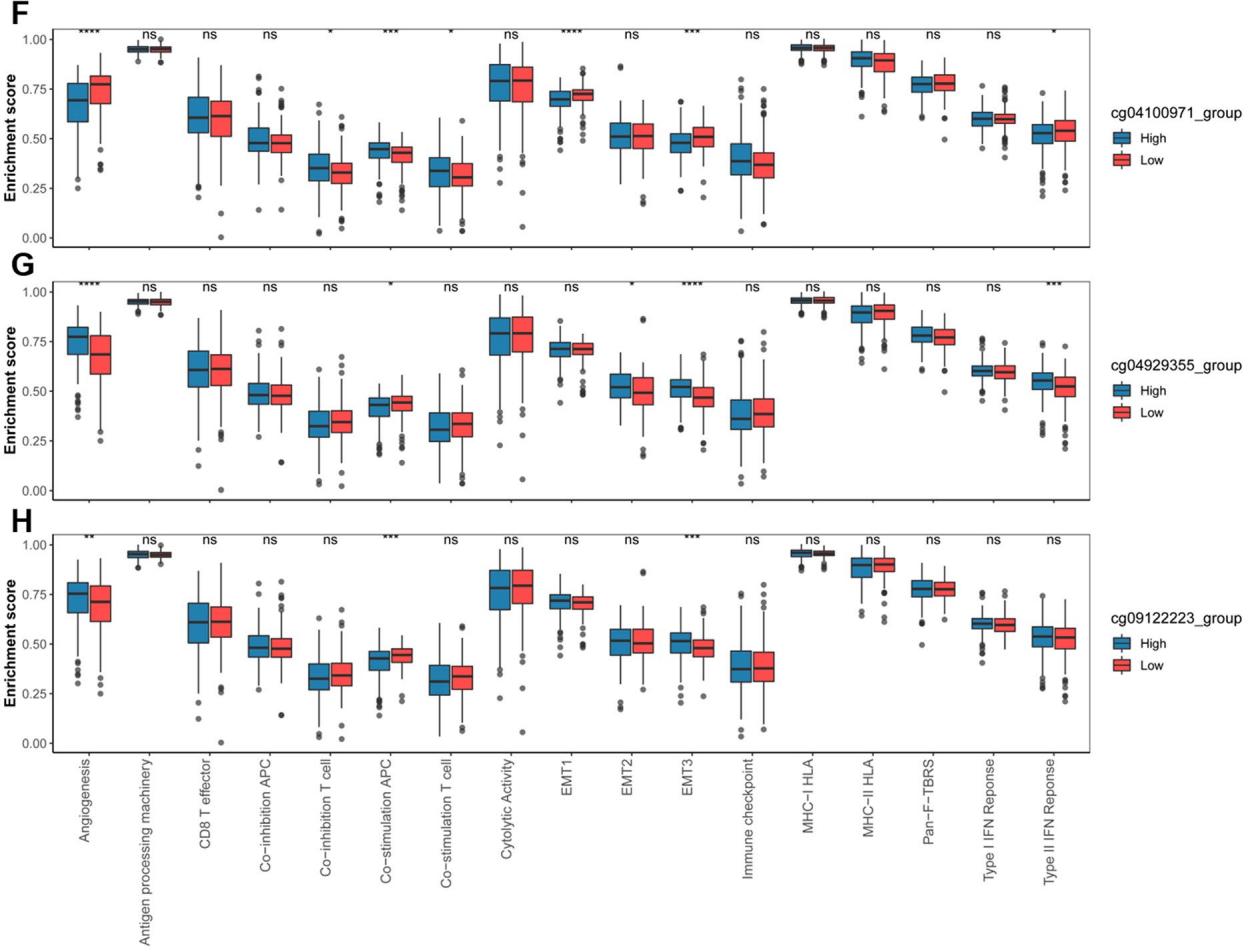
IL18 expression and promoter methylation were correlated with immune cell infiltration in RCC**.** **A**. The correlation heatmap of IL18 expression and its differentially methylated sites with 23 types of immune cells in the TCGA RCC cohort, only statistically significant (*P* < 0.05) were shown in correlation coefficients. **B**–**H**. Relative enrichment scores of 17 immune-related pathways in the high and low groups according to the median of IL18 expression and the methylation of cg04100971, cg04929355, cg05687149, cg09122223, cg11304234 and cg26534425 respectively

As it was reported that DNA methylation was closely related to tumor microenvironment, we further investigated the potential of IL18 and promoter methylation and expression in immune regulation. We analyzed the correlation of IL18 expression and promoter methylation in 23 immune cell lines including B cells, as well as CD4 and CD8 T cells. We found that high IL18 expression was associated with higher infiltration of immune cells (Fig. 7A). We also found that the methylation levels of the IL18 promoter were clearly associated with immune infiltration of immune cells. Among all the examined sites, we found cg04100971 hypermethylation and cg0912223 hypomethylation were significantly correlated with infiltration of immune cells (Fig. 7A). To further analyze the key immune-related pathways, we chosen 17 biological pathways, analyzed the correlation between them and IL18 expression and promoter methylation. We found that lower expression of IL18 was correlated with a higher angiogenesis score (Fig. 7B), meaning that patients with low IL18 expression were probably sensitive to anti-angiogenesis therapy. Besides, lower IL18 expression was also related to lower scores of processing machinery, CD8 T effector, antigen cytolytic activity, and MHC-HLA, which indicates the infiltration of lower immune cells in the tumor microenvironment. Also, higher IL18 expression was significantly related to higher co-inhibition APC, co-stimulation APC, co-inhibition T cell signature immune checkpoint activity and EMT3 (Fig. 7B). All these showed that tumors with high IL18 expression were abundant in the infiltration of the immune cells, and they also had stronger immune checkpoint signature resulting in an immunosuppressive microenvironment. Next, we analyzed the relation of IL18 promoter methylation with these 17 immune-related pathways (Fig. 7C–H). The cg11304234 and cg26534425 hypomethylation were associated with lower angiogenesis, but higher co-inhibition T cell signature, immune checkpoint activity, co-stimulation APC (Fig. 7C, D), which were consistent with our results in IL18 expression. These results indicated that high IL18 expression and promoter hypomethylation were positively correlated with immune cell infiltration, that contains high enrichment of immune checkpoint molecules, which may sensitively respond to immune checkpoint inhibitors. Therefore, IL18 expression and promoter methylation were the potential molecular biomarkers to predict the responses of RCC patients to immune checkpoint inhibitors therapy.

### IL18 expression and promoter methylation were associated with the expression of key immunomodulators in RCC

**Fig. 8:**
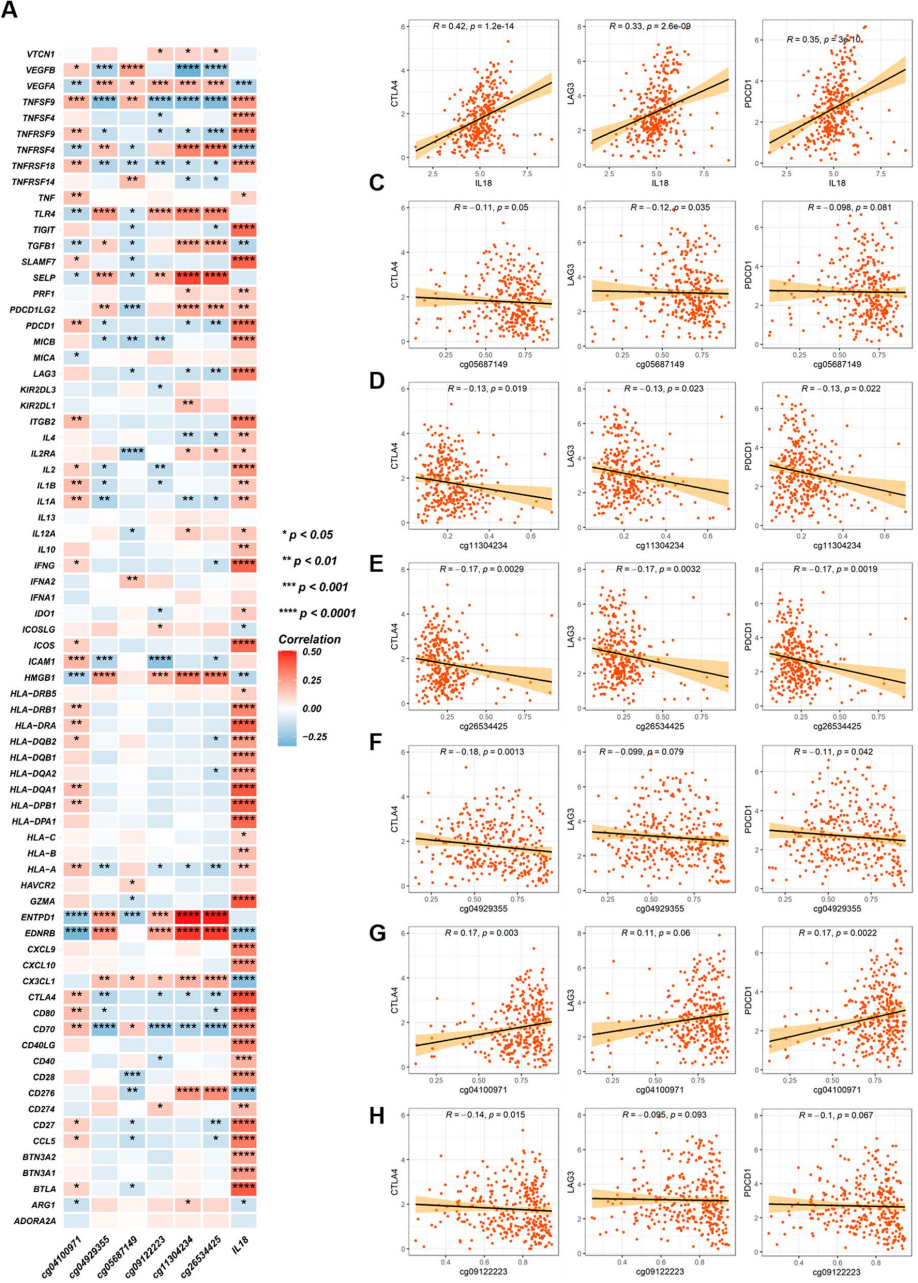
IL18 expression and its promoter methylation were associated with the expression of the key immunomodulators in RCC. **A**. The correlation heatmap of IL18 expression and the six methylated CpG sites with the key immunomodulators in the TCGA RCC cohort, only statistically significant (*P* < 0.05) are shown correlation coefficients. **B**–**D**. The correlation of JAK3 expression, cg04100971, cg04929355, cg05687149, cg09122223, cg11304234 and cg26534425 methylation with immune checkpoint molecules CTLA4, LAG3, PACD1, respectively

It was reported that tumor immune microenvironment could be regulated by various membrane proteins and cytokines. To clarify the relationship of IL18 expression and promoter methylation with the key molecules participating in the anti-tumor immune responses, we explored the relationship between IL18 expression and promoter methylation and 74 key immunomodulators using the TCGA cohort. We found that high IL18 expression and hypomethylation of cg04100971 and cg0912223 were correlated with the expression of most immunomodulators (Fig. 8A). Based on the different therapeutic effect of immune checkpoint inhibitors on various tumors, we analyzed the connection of IL18 expression and cg04100971, cg05687149, cg04929355, cg09122223, cg11304234 and cg26534425 methylation with three immune checkpoint molecules (CTLA4, LAG3, and PDCD1), using expression data from the TCGA dataset. We found that IL18 expression was significantly positive related to the expression of CTLA4, LAG3, and PDCD1 (Fig. 8B), whereas cg05687149, cg04929355, cg11304234 and cg26534425 methylation were negatively associated with expression of CTLA4, LAG3, and PDCD1 (Fig. 8C–F). These results showed that IL18 expression and its promoter methylation were related to the immune suppressive microenvironment in RCC.

## Discussion

Increasing studies have shown that abnormal DNA methylation is involved in the occurrence and development of various diseases and tumors. It has been reported that the level of methylation in tumor cells plays a crucial role in anti-tumor immunity and the efficacy of immunotherapy. The detection of DNA methylation levels has gradually become an effective method for tumor diagnosis and prognosis prediction. Nowadays, there are more and more studies focusing on the effect of DNA methylation on immunotherapy and prognosis of RCC. In this study, we found that IL18 promoter was generally aberrantly methylated in RCC tumor tissues compared to the normal tissues. We confirmed that IL18 is highly expressed in RCC tumor, and high IL18 expression is associated with different pathological stages and poor prognosis in RCC patients (Fig. 1A–F) Then, we identified six significantly differentially methylated CpG sites: cg04100971, cg04929355, cg05687149 cg09122223, cg11304234 and cg26534425, most of which were significantly hypomethylated in tumor tissues except cg04100971 (Fig. 2A–C). It has been reported that abnormal methylation of specific genes can lead to abnormal gene expression. Hypermethylation of specific genes often leads to gene silencing, whereas, hypomethylation in gene promoters usually results in gene activation because of more open chromatin structure [20].

In line with this, the CpG sites (cg04929355, cg05687149 cg09122223, cg11304234, cg26534425) in this study are located in the IL18 promoter and their methylation levels are negatively correlated with IL18 expression. Given that the cg05687149 sites was hypermethylated in RCC tumor (Fig. 2A–C), which was also not related to IL18 expression (Fig. 4A) but associated with the immune cell infiltration and immune suppressive microenvironment in RCC (Figs. 7A, E and 8A, G), we proposed that CpG sites located in different regions of IL18 promoter might be bound by different regulator such as transcription factors and chromatin remodelers, which could affect the expression of IL18 and its biological functions. This warrants further investigation in the future. These results suggest that aberrant IL18 promoter methylation may result in its higher expression in RCC tumor. To further explore the clinical significance of IL18 promoter methylation, we analyzed the association of IL18 promoter methylation with RCC patient survival. By a Kaplan–Meier survival analysis, we found that hypomethylation of IL18 promoter was associated with shorter survival (Fig. 5A–F), which was consistent with our finding that IL18 promoter methylation (except cg04100971) was negatively correlated with IL18 expression. Our results are consistent with the previous assumptions. Indeed, most of the hypomethylated sites we analyzed were associated with poor overall survival (expect cg04100971) (Fig. 5A–F). We also found that among these sites, cg04929355 was the most statistically significant one (*P* < 0.0001) (Fig. 5B). Based on our above results, we propose that cg4929355 is the most notable prognostic predictor for RCC patients. Regarding the cg04100971 site hypomethylation was related to favorable prognosis (Fig. 5A), this paradox may be because this site was already highly expressed in RCC tumor. We also found that IL18 expression was different in renal cells with different pathological grades and stages. The more advanced the grade, the higher the expression of IL18 was. Additionally, the IL18 promoter methylation was different in different pathological grades and stages, and among these sites cg04929355 was significantly hypomethylated in all advanced pathological grades and stages (Fig. 3B, H). These results showed that cg04929355 methylation may be a prominent prognostic predictor for RCC patients.

As is reported, DNA methylation plays an increasingly important role in cancer diagnosis and prognosis prediction, and DNA methylation markers also appear to be accurate and promising predictors of patient outcomes in immunotherapy [25]. IL18 is well known as a crucial member of IL1 family, which plays an important role in immune regulation. We wondered whether IL18 expression and its’ promoter methylation could also be the predictor of immune cell infiltration in RCC. To test our conjecture, we used the series of DNA methylation biomarkers located in the promoter of immune-related genes identified by other researchers [24], which can predict the immune cell infiltration and responses to immune checkpoint inhibition in cancers. We also analyzed the connection of IL18 expression and its promoter methylation to the 23 immune cell lines in the tumor microenvironment. We found that IL18 expression was significantly positively correlated with most of the analyzed immune cells, especially CD8 + T cells, in the TCGA RCC database (Fig. 7A). Besides, we found that IL18 promoter hypomethylation was also positively correlated with most of the analyzed immune cells. It has been reported that high levels of tumor CD8 + T cell infiltration were associated with a worse prognosis in RCC patients [26], which was consistent with our findings that high IL8 expression and promoter hypomethylation were positively related to the immune cell infiltration but predicted a worse prognosis. This may be due to the increase of inhibitory immune checkpoint molecules, which lead to the inactivation of anti-tumor immunity [27]. Therefore, we analyzed the correlation of IL8 expression and promoter methylation with 74 key immunomodulators using data from TCGA dataset. The results came out that IL8 expression was significantly correlated with the expression of most of the immune checkpoint molecules. We also found that IL8 promoter hypomethylation was correlated with some of the immune checkpoint molecules such as CD70, TNFRSF18, CTLA4, LAG3, and PDCD1 (Fig. 8A–H.) These results revealed the relationship of IL8 expression and its promoter methylation. And the important role between IL8 expression and its promoter methylation in overall survival, clinicopathological characteristics, and immune cell infiltration in RCC patients.

In addition, the main database used in our study was TCGA, and in this cohort, HumanMethylation450 Bead Chip beads, were used to analyze the methylation levels.

As it didn’t cover all CpG sites of human genomic DNA. There can be other methylation sites yet to be identified as better candidates to predict prognosis and immune infiltration. Although we have proved that the IL18 expression and its methylation were associated with immune cell infiltration in RCC, our study did not validate with a cohort which received immune checkpoint inhibitors therapy. The regulation of tumor immunity by genes and epigenetic modifications is a complex process, which requires more physiological and biochemical experiments, especially the in vivo experiments to verify. Therefore, we will do more experiments to clarify the mechanism by which IL18 and its promoter site methylation regulates renal cell immune infiltration and immune checkpoint inhibition. Based on our findings of the significantly positive correlation of IL18 expression and its promoter methylation with multiple immune checkpoint molecules (LAG3, CTLA4), it may a breakout to investigate whether IL18 has the ability to regulate the expression of these immune checkpoint molecules. If so, combination of IL18 inhibitors with immune checkpoint inhibitors may be a new strategy in RCC treatment. Taken together, our study confirmed that IL18 promoter methylation was associated with IL18 expression, clinicopathological characteristics, and overall survival in RCC. Moreover it is related to immune cell infiltration and expression of immune checkpoint molecules. We provided new evidence that IL18 promoter methylation may serve as molecular biomarkers for predicting prognosis and responses to immune checkpoint inhibitors, which may benefit the development of immunotherapy in RCC patients.

## Conclusions

In summary, by using bioinformatics methods, we have here identified IL18 promoter methylation may serve as molecular biomarkers for predicting prognosis and responses to immune checkpoint inhibitors in RCC. We propose future studies to fully explore the significance of IL18 promoter methylation in responses to immune checkpoint inhibitors by an experimental approach.

## Methods

### TCGA database

The RCC related clinical information, gene expression and methylation data were downloaded from the Cancer Genome Atlas Data Portal (TCGA, http://cancergenome.nih.gov/). We also download the RNA-seq data expressed in parts per million transcripts (TPM) from TCGA database, and used R package (edgeR) and R version 4.0.3 software to normalize and process relevant data. We used the R package ‘chAMP’ to analyze the methylation data. Analyzing the differential methylation site, obtained from Infinium Human Methylation450 BeadChip beads. Methylation and transcriptome analysis were carried out on 317 tumor tissues. The clinical pathological information of TCGA patients was supplemented in Additional file 2: Table S1.

### GEO datasets and CPTAC

The gene expression data and methylation data were from the Clinical Proteomic Tumor Analysis Consortium (CPTAC) of the National Cancer. The same method used in TCGA database was used to analyze the expression and methylation. The other methylation data of GSE70303 and GSE105260 were downloaded from the gene expression comprehensive (GEO) database to examine the methylation of differential IL18 promoters.

### Methylation analysis

The related methylation data were download from TCGA and CPTAC database to obtain more information of methylation. We used the Beta-value to assess the methylation levels, and analyze the CpG sites in IL18 which were target by the Infinium Human Methylation450 BeadChip beads. We prefer the R package ‘limma’ to normalize the standard of adjust *P* value < 0.05 and log (Fold Change) > 0.05, to analyze the differential methylation sites.

### Cell lines and cell culture

The human RCC cell lines 786-O and ACHN were obtained from American Type Culture Collection (ATCC, Manassas, VA) and cultured in RPMI-1640 (Invitrogen, Carlsbad, CA). All cell lines were maintained in an incubator with a humidified atmosphere of 95% air and 5% CO2 at 37℃.

### RNA extraction and RT-qPCR

Total RNA was extracted using RN001 RNA Quick Purification kit from ESscience (RN001) following the instructions. cDNA was synthesized using RT001 Fast Reverse Transcription kit from ESscience (RT001) following the instructions. qPCR was performed using ChamQ SYBR qPCR Master Mix from Vazyme (Q311-02) following instructions. β-actin was used as internal control. The primers were displayed as follows. IL18-F:GATAGCCAGCCTAGAGGTATGG,

IL18-R:CCTTGATGTTATCAGGAGGATTCA;

β-actin-F:CGTCTTCCCCTCCATCGT,

β-actin-R:GAAGGTGTGGTGCCAGATTT.

### Genomic variation analysis (GSVA) to assess immune cell infiltration in the tumor microenvironment

We used the R-pack "GSVE" and a single-sample gene collection and enrichment analysis (ssGSEA) algorithm to analyze the invasion and biological pathways of immune cells and calculate the relative expression levels of immune infiltrating cells in the tumor microenvironment. All the gene used in evaluating the immune cells infiltrated in each tumor microenvironment comes from Lang and Zhou’ study [28], (Additional file 2: Table S3), which contains 23 human immune cell subtypes. And the enrichment fraction calculated using ssGSEA was the relative abundance of infiltrated immune cells in each tumor microenvironment in each sample. Relative abundance of tumor microenvironment cells in each sample of TCGA was supplemented in Additional file 2: Table S4.

### Analysis of the IL18 expression/promoter methylation in relation to other biological processes

We utilized the gene sets constructed by other researchers, which related to some biological processes or tumor immune microenvironment. These gene sets contains may different genes such as immune checkpoint, CD8 T effector, Epithelial mesenchymal transition (EMT) markers, IFN reaction, and so on[24, 29], (Additional file 2: Table S5). We tested the enrichment scores of each gene expression and biological process of IL18 promoter methylation and expression in low and high groups, respectively. Relative enrichment scores of immune signatures in TCGA were supplemented in Additional file 2: Table S6.

### Statistical analysis

In this study, SPSS (version23.0), GraphPad prism (version 8) and R(version 4.0.3) were used to analyze the data. We also reckoned the correlation coefficient by Spearman's rank correlation (Spearman's R), and used T test or Mann Whitney test to compare the differences between the two groups. We used the critical value, calculated by R software package ‘survminer’ for the survival analysis. We used Kaplan–Meier survival analysis to analyze the relationship between IL18 expression or promoter methylation and overall survival in RCC. Log rank test was used to determine the significance of the difference. All statistical *P* values were positive or negative, with P < 0.05 as statistically significant *P < 0.05, **P < 0.01, ***P < 0.001, ****P < 0.0001).

## Supplementary Information

**Additional file 1.** The supplementary figures in this study.

**Additional file 2.** The supplementary tables in this study.

## Data Availability

All processed data generated or analyzed in this study are included in the additional files.

## Abbreviations

IL18: Interleukin
IFN: Interferon
TNFα: Tumor necrosis factor
COX2: Cyclooxygenase2
VEGF: Vascular endothelial growth factor
NLRP3: NLR family pyrin domain containing 3
GM-CSF: Granulocyte-macrophage colony-stimulating factor
CTLA4: Cytotoxic T-lymphocyte antigen 4
LAG3: Lymphocyte-activation gene 3
PDCD1: Programmed cell death protein 1
