# Supplementary material for: The prognostic value and immune correlation of IL18 expression and promoter methylation in renal cell carcinoma
Source: Clin Epigenetics. 2023 Jan 28;15:14. doi: 10.1186/s13148-023-01426-8 (PMC9883904; doi:10.1186/s13148-023-01426-8)
Supplement: Supplementary file 1 — Additional file 1. The supplementary figures in this study. [file 13148_2023_1426_MOESM1_ESM.docx]

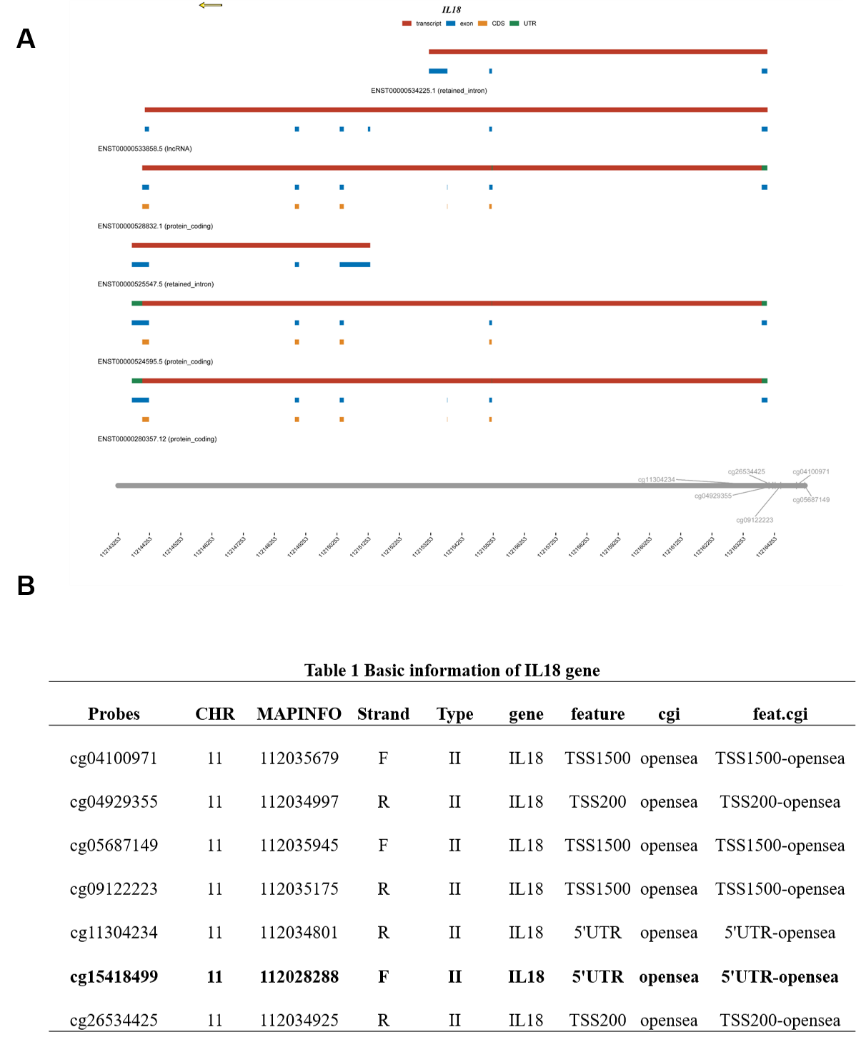


**Figure S1. Basic information of IL18.**

1. The schematic diagram of IL18 structure. B. Basic information of the probes


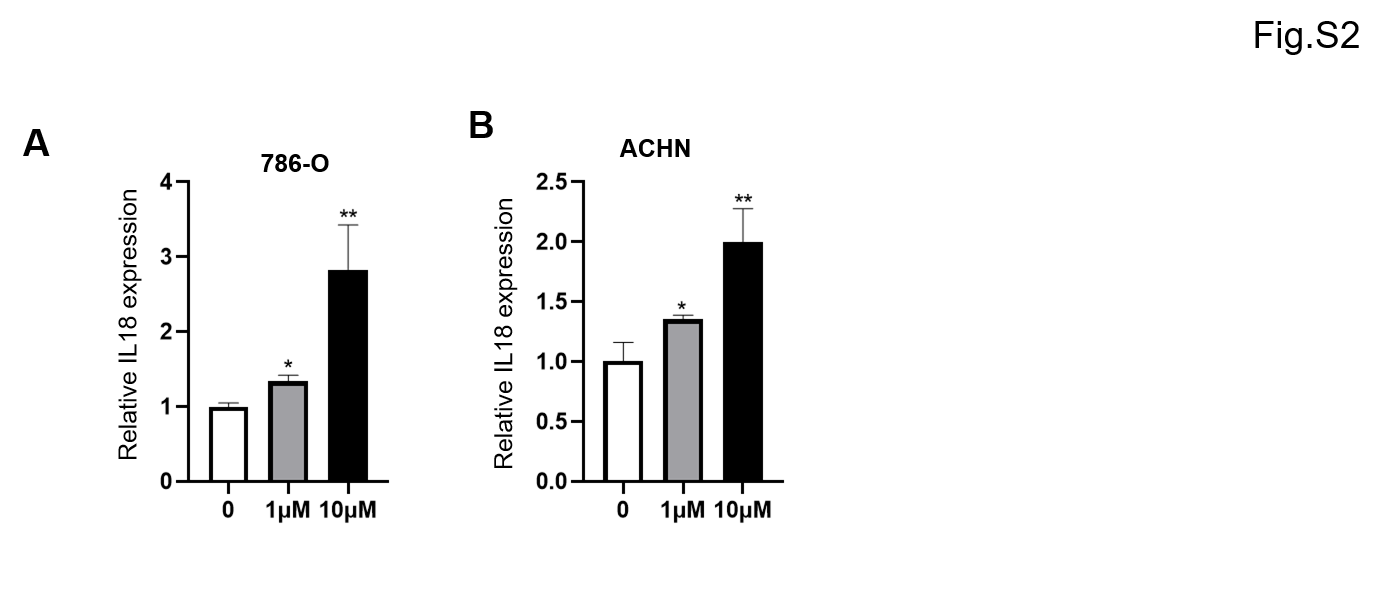


**Figure S2. Relative IL18 mRNA expression of RCC cells after treatment with 5-Azacytidine.** A. Relative IL18 mRNA expression of 786-O after treatment with 1μM or 10μM 5-Azacytidine, respectively. B. Relative IL18 mRNA expression of ACHN cells after treatment with 1μM or 10μM 5-Azacytidine, respectively.
